# Lifestyle Interventions through Participatory Research: A Mixed-Methods Systematic Review of Alcohol and Other Breast Cancer Behavioural Risk Factors

**DOI:** 10.3390/ijerph19020980

**Published:** 2022-01-16

**Authors:** Jessica A. Thomas, Emma R. Miller, Paul R. Ward

**Affiliations:** 1 Discipline of Public Health, Flinders University, Adelaide, SA 5001, Australia; emma.miller@flinders.edu.au; 2 Centre for Health Policy Research, Torrens University, Adelaide, SA 5000, Australia; paulward0161@gmail.com

**Keywords:** breast cancer, alcohol, lifestyle modification, primary prevention, participatory research

## Abstract

Breast cancer is one of the most frequently diagnosed cancers in women globally. Sex and advancing age represent the dominant risk factors, with strong evidence of alcohol as a modifiable risk factor. The carcinogenic nature of alcohol has been known for over twenty years; however, this has failed to translate into significant behavioural, practice, or policy change. As a result, women have not benefitted from this research and, by extension, have been exposed to unnecessary breast cancer risk. Participatory research presents a solution to research translation in public health through the collaboration of impacted populations with academics in research. This systematic review examines peer-reviewed research studies where participants were involved in the research process and the outcomes related to breast cancer prevention (either alcohol or broader lifestyle modification). Seven of the eight studies reported positive effects, and the collaboration between academic researchers and impacted populations may have supported positive outcomes. Women were receptive and responsive to participatory approaches, and their participation is important to address socially entrenched behaviours such as alcohol consumption. Participatory research presents opportunities for future interventions to improve (or address) modifiable risk factors for breast cancer.

## 1. Introduction

Breast cancer is one of the most frequently diagnosed cancers in women, with 2.3 million new cases diagnosed globally in 2020 [1]. Population projections for future breast cancer incidence predict a significant increase due to aging populations [2]. Aging populations can increase demand on healthcare resources with a lower proportion of the population in the tax-paying workforce [3]. There is a strong need for sustainable and effective preventative interventions in breast cancer. This review synthesizes the literature to understand the effectiveness of lifestyle interventions to inform future research. The mechanisms, the approaches to conducting research with communities through participatory research, will be examined to inform future primary prevention of breast cancer.

Carcinogenesis is a multistep process involving several cellular changes over time, and lifestyle patterns play a key role in primary prevention [4,5]. An integrated pattern of behaviours maintained over time, a way of life, limiting alcohol use, incorporating physical activity, and healthy eating present the opportunity for the greatest risk reduction of breast cancer [6]. Designing effective risk reduction interventions requires knowledge of previous research on the outcomes and the key mechanisms of perception, knowledge, risk, and attitudes which influence behaviours [7,8].

Evidence suggests the potential for breast cancer risk reduction through lifestyle modifications. Women drinking heavily have a 60% excess risk for breast cancer relative to nondrinkers [9]. However, consumption of alcohol at low levels previously considered “safe” has been found to significantly elevate cancer risk [10]. The World Health Organization recommend that people should limit alcohol consumption to reduce cancer risk [11,12].

Whilst the carcinogenic nature of alcohol has been known for over 20 years, this has failed to translate into significant practice and policy change [13]. As a result, women have not benefitted from this research and have been exposed to unnecessary breast cancer risk. The involvement of the impacted population and decision-makers with researchers has been suggested as a solution to the common research translation failure [14]. Participatory action research (PAR) attempts to bridge the gap between research and practice by “creating conditions that facilitate people’s control over the determinants of their health” [15]. A key difference from traditional research is that PAR works with the population experiencing the health issue and stakeholders, who in turn take actions to improve health [16]. To achieve meaningful and sustainable change, PAR requires a shift in power and a reconfiguration of the roles of the investigators and the participants moving from passive research participants to partners in the research process [17]. 

PAR has proven beneficial in research translation with women across a breadth of preventative health areas, including cardiovascular disease [18], diabetes [19], and physical activity [20]. Effective interventions in these health areas have overlapping lifestyle risk factors with breast cancer, indicating that the mechanism of PAR may be beneficial to primary breast cancer prevention. 

Secondary prevention of breast cancer through screening interventions and cancer control have found the research approach of PAR beneficial to research outcomes. PAR studies seeking to improve health equity found that women from the intervention group were 10 times more likely to undertake breast cancer screening than the control group who received written educational materials [21]. Cancer control has benefited from PAR through patients contributing to the improvement of healthcare services [22]. The study found that PAR enabled the voices of people with cancer to be heard, which improved problem-solving and supported equity in the redesign of health services. Similarly, a review of PAR and health services found that the advantages of PAR were the creative and imaginative solutions generated due to the study population’s understanding of the unique patient experiences [23]. 

Despite the potential shown in related research areas, no reviews of literature using PAR in breast cancer prevention and, more specifically, in alcohol use, have been previously published. Previous systematic reviews of PAR with young people [24,25,26] have highlighted important methodological learnings to inform future interventions. The purpose of this research is to inform future primary-prevention breast cancer interventions. 

Whilst the research areas of breast cancer and alcohol are well established, the literature on participatory research approaches in this field is small and emerging. New approaches to research are worth exploring, particularly given the lack of research translation in alcohol-related breast cancer risk reduction. Given the limited research available, a broader scope for this review incorporating research on alcohol use utilizing participatory approaches has been included, even though it may not be breast cancer- or gender-specific. The diverse literature could still provide important methodological PAR learnings of how working with impacted communities constrained or contributed to intervention effectiveness. Community-based interventions are the nature of PAR, where researchers work with communities; therefore, the study population is likely to be diverse and not gender-specific. Whilst broader breadth of population is likely, studies will only be relevant if they incorporate female participants. Empirical studies reporting on breast cancer lifestyle risk factors where the study population had active involvement in at least one stage of the research process will be analysed to ascertain the following: (1) How effective are PAR lifestyle change interventions; (2) What enabled and challenged interventions? 

## 2. Materials and Methods

### 2.1. Design

**Figure 1:**
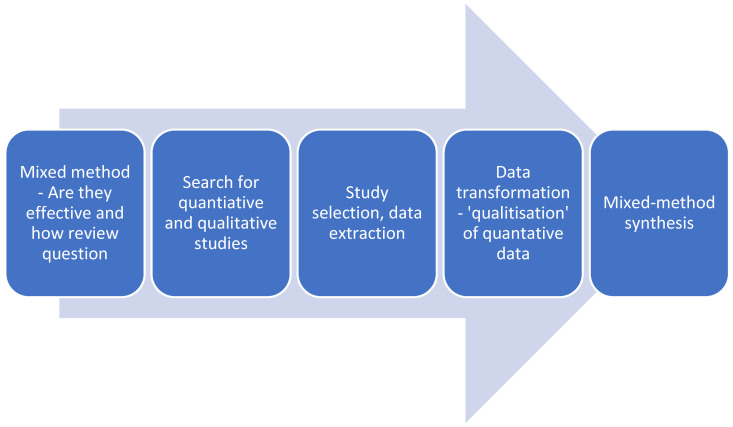
Figure adapted from convergent integrated approach [29].

A mixed-methods systematic review of primary studies utilising a convergent integrated approach was completed. The Joanna Briggs Institute approach to mixed-methods systematic reviews to synthesis was followed [27]. Figure 1 outlines the steps undertaken in the convergent integrated synthesis. The review follows PRISMA reporting guidelines [28] and the protocol is listed on PROSPERO (CRD42019135545).

### 2.2. Search Strategy

Consultation with experts in the field and existing systematic reviews [24,30,31,32] informed the search strategy (Appendix A). The guidelines from the Cochrane Collaboration Handbook on *Systematic Reviews of Health Promotion and Public Health Interventions* approach to systematic review planning and searching was followed [33]. A sensitive search to capture participatory research approaches across several disciplines was formed. Multiple iterations of the search were performed in PubMed and Ovid MEDLINE in consultation with an expert research librarian. The additional databases of Web of Science, ProQuest, and Scopus were searched by the librarian to identify additional studies that the original search may have missed. This additional search did not locate any relevant articles that had not already been identified. After the comprehensive search strategy was finalised, the final search was conducted on 18 May 2021, in Ovid PsycInfo, MEDLINE, and Ovid EMCARE. On the 9 June 2021, the search was tailored for and run in Cochrane Library.

### 2.3. Eligibility Criteria

The research questions sought to understand the effectiveness and the mechanisms of previous interventions with a participatory approach. A mixed-methods systematic review can utilize both positivist and constructivist types of knowledge and present a more comprehensive picture of the evidence for future decision-makers [34]. A broader synthesis of the evidence, methodologically inclusive of qualitative, mixed-method, and quantitative studies, was completed [35,36]. To gain the methodological depth required to address review objectives, only primary research articles were included. Secondary sources, such as systematic reviews and other types of literature reviews, had their reference lists manually searched for eligible primary studies, as were the reference lists of all included studies. 

Experimental studies from a range of study designs were included (randomized trials, controlled trials, and quasi-experimental designs) which aimed to assess the impact of lifestyle interventions. Whilst the prevalence of breast cancer is far greater in females, breast cancer does occur in men, and alcohol consumption increases the risk of breast cancer in both males and females [37,38]. Therefore, studies with the participation of males and females were included. Whilst there is evidence of overweight and obesity as a risk factor for breast cancer, interventions are highly likely to include the primary influencing behaviours of eating and physical activity. For this reason, obesity was not specifically searched, due to coverage of the comprehensive nutrition and physical activity search strings. Studies were considered if alcohol was reported by gender or other lifestyle breast cancer prevention interventions reporting on (smoking, physical activity, diet) with or without alcohol to gain broad insight into participatory research in breast cancer prevention.

All searches were limited to 2008–2021 for three reasons: current best practice, relevance, and technological change. The review aimed to synthesise the literature based on the assumption that recent interventions represent current best-evidence practice. A similar approach was used by a systematic review of alcohol interventions and young people [39]. The search is focused on recent literature, as the recognition of alcohol as a risk factor for breast cancer was established in 2007 through a comprehensive review [40]. In 2010, for the first time, alcohol was recognised as a priority for poor health globally and a landmark global strategy on the harmful use of alcohol was created [41]. In 2011, the Cancer Council of Australia released a position statement and for the first time which recommended that alcohol should be limited or avoided to reduce cancer risk [42]. Thirdly, with the rise of digital technologies, the ways in which people communicate and access information has changed [43]. Portable mobile devices such as smartphones and tablets, with greater social capabilities, are omnipresent [44]. These social capabilities of portable devices and access to social media has changed the way people engage and collaborate, which is important for understanding participatory action approaches both in terms of implementation models and technology-supported interventions; the use of technology to communicate health messages through social media is an example. Therefore, for each of these three reasons, literature within the last decade will be more relevant to achieving the review objectives.

### 2.4. Study Selection

**Figure 2:**
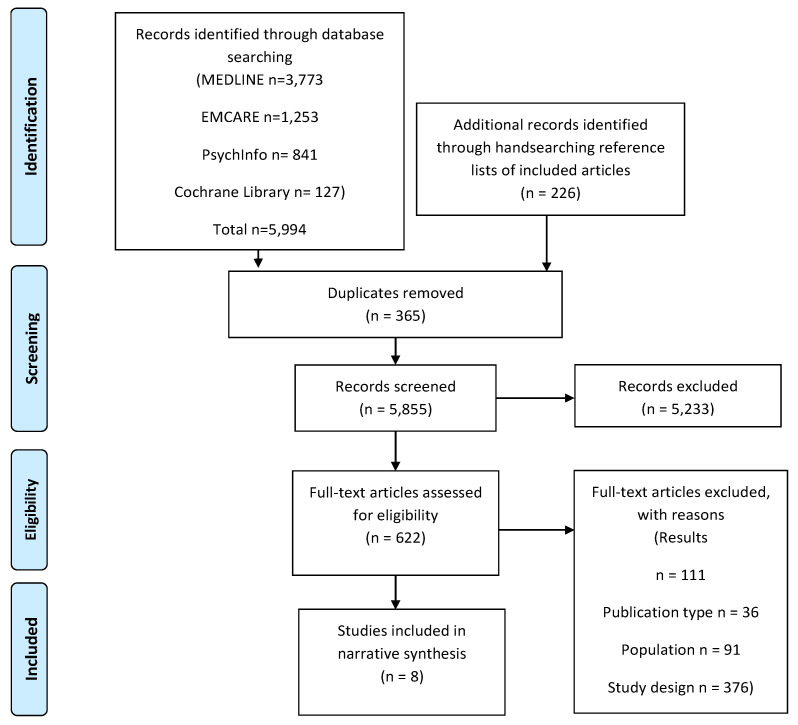
Search result PRISMA diagram.

The identified articles were collated in reference management software and duplicates were removed. The titles and abstracts were screened against the eligibility criteria. The studies meeting the eligibility criteria advanced to full-text screening and the PAR principles criteria (defined in the intervention category of Table 1) was applied. This criterion was only applied at full-text screening as full details of study methods were needed to accurately assess the citations. Disagreements at the full-text screening stage were resolved by discussion, and consensus was reached. The quality appraisal was completed by two independent reviewers (JT and PW) using the JBI critical assessment tools for qualitative studies, quasi-experimental studies, and randomized controlled trials [45]. The process is presented through the PRISMA diagram, Figure 2. 

### 2.5. Data Extraction

A data extraction form was designed based on the protocol and included aim, behaviours targeted, theoretical frameworks, study design (population, setting, intervention details), participation of the study population aspects, data collection methods, results, discussion, and limitations. The authors’ reflections on key learnings/barriers were extracted from the discussion and limitations sections for information on the intervention enablers, challenges, and mechanisms.

### 2.6. Analysis

The nature of the review questions demanded an evidence synthesis of mixed-methods studies examining a broad range of behavioural outcomes. The lack of homogeneity prevented a quantitative meta-analysis. The JBI manual for evidence synthesis (2020) was used to guide the mixed-methods synthesis [27]. The review question required both quantitative and qualitative research designs to address what works and how; therefore, the convergent integrated approach was most appropriate [46]. An integrated approach, where the data was extracted and then the quantitative data was transformed into “qualitised” data through a narrative interpretation of the results, was completed [46]. This is preferred to the alternative approach of transforming the qualitative data into numerical values which is more prone to errors [27]. The extracted and transformed data was thematically analysed according to Braun and Clarke’s principles [47]. The data regarding effectiveness and the authors’ discussion on what contributed to or limited the study was coded inductively and then collated into themes through a process of repeatedly examining the data to identify categories of similar meaning. Lastly, categories were grouped and synthesised according to the review objectives [27].

## 3. Results

### 3.1. Methodological Adequacy

Selection bias, retention rates, allocation bias, and blinding are discussed in this section. Studies with less than a 100% consent rate have the potential for selection bias of participants. The studies’ response rates, defined as eligible participants consented to participate, were 85% [48], 64% [49], 47% [50], 35% [51], and 24% [52]. The response rates were not provided by three studies reducing ability to assess selection bias [53,54,55]. As seen in Table 2, a randomized control trial design was used by four studies increasing methodological rigor by minimising the risk of allocation bias and providing baseline differences in comparison groups [48,50,52,53].

High retention rates were achieved by two studies, reported as 98% [52] and 96% [49], reducing the selection bias in the analysis. Moderate retention rates were achieved at 79% [48] and 68% was reported by two studies [50,51].The Thai-based community cluster RCT study used a second random sample for the follow-up survey from the intervention and control communities, and therefore retention rates were not applicable [53]. Loss to follow-up was not appropriate to examine for the qualitative studies [54,55].

Double blinding was used by one alcohol consumption intervention study where participants and outcome assessors were blinded to the group allocation [51]. In community-based research, the blinding of participants and facilitators delivering the program is less feasible as they have contact with each other, however the blinding of outcome assessors is more practical. The outcome assessors were blinded in the peer mentoring of individuals with a traumatic brain injury RCT study [48]. Blinding was not used in any form by three RCT studies increasing the risk of measurement bias [50,52,53,56]. 

### 3.2. Intervention Characteristics and Effectiveness

The literature was dominated by alcohol-use interventions (as seen in Table 2) with seven of the eight studies, the exception being a study focused on nutrition and breast cancer prevention [49]. However, the literature was diverse in terms of study designs, settings, and populations, reducing the ability to interpret effectiveness. Five interventions were conducted in the United States of America (US) [48,49,50,51,52], and three in Thailand [53,54,55]. Two of the articles came from a related Thailand-based study [54,55]. 

Six of the eight included studies used a peer-led health promotion approach defined as “the teaching or sharing of health information, values and behaviours by members of similar age or status groups” [57]. The facilitators shared health information and were of similar age, gender, cultural background, and from the same community as the study participants.. The peer-led approaches were found to be effective in all studies utilizing this approach [48,49,50,51,54,55]. The remaining studies sought to reduce alcohol use through a mobile application storytelling intervention [52] and through structural factors through a multisector regional intervention [53].

Three studies used a community coalition approach [53,54,55]. One study did so without a peer component and attempted a policy focus to reduce alcohol use [53]. The coalitions met regularly with researchers and the study population was involved in several stages of the research process from problem identification, defining research objectives and developing the intervention [53,54,55]. 

In the alcohol-focused interventions, six of the seven studies found significant reductions in alcohol use [48,50,51,52,54,55]. The single study which did not achieve positive results was the community cluster RCT, where alcohol use remained high in control and intervention communities. Only minor changes were present from baseline, and the overall study reported a null effect [53]. The nutrition and breast cancer prevention intervention reported positive results, with improvements in breast cancer knowledge and increased fruit and vegetable dietary intake [49]. 

An RCT utilising empowering storytelling through animations on a tablet-based device found reductions in alcohol use [52]. The mHealth intervention was run in community health clinics with a user interface and animations which were designed in consultation with the study population to deliver risk-reduction health messages about several behaviours, including alcohol use [52]. A quasi-experimental study incorporating cultural values to support wellness reported a reduction in alcohol consumption [51]. Women who consumed moderate to high levels of alcohol prior to the intervention reported greater substantial absolute decrease than lighter drinkers [51]. Participants from the two experimental groups (one group with health education and one with health education and cognitive-behavioural skills building) found the same levels of increase in self-efficacy to reduce their drinking and an increase in self-esteem [51]. 

One study compared people with a traumatic brain injury (TBI) and their significant others to control groups [48]. The study found both peer mentoring groups to have significant improvement in enhanced quality of life and ability to cope with life’s challenges and reductions in alcohol use compared to the control group [48]. Likewise, the studies based in Thailand found that the peer model was effective in empowering participants to share knowledge regarding alcohol use, and assisted families with members with a problematic alcohol consumption to reduce alcohol use [55]. 

### 3.3. The Involvement of the Study Population in the Research

A key and distinguishing feature of participatory research approaches is the empowerment of the impacted population. PAR is a methodology in which researchers work with communities to support health-affirming action [16]. Diverse approaches and varying degrees of “working with” mechanisms were used. In three studies [48,49,50], the involvement of the communities in the research process appears to be motivated by convenience and feasibility rather than a commitment to the equity principles of PAR ideology, which seeks to increase the power of those impacted to take actions to improve their health [14]. The two peer-mentoring program interventions [48,50], the college-based and TBI communities alcohol interventions, had lower levels of involvement of the impacted population in the research process. Peer mentors were trained to deliver a standardized intervention, and members of the study population contributed to the design of a questionnaire to evaluate the TBI communities intervention [48]; the study population of students were involved in the analysis phase through coding video-recorded peer mentoring sessions [50]. Coding was completed for the level of empathy shown by mentors, number of reflections made by the participants, and the number of open and closed questions asked by peer mentors. High levels of inter-rater reliability among the six college student coders was found [50]. The study did not discuss how the mechanism of PAR contributed to the research outcomes. In contrast the authors state that the principles of PAR underpinned the Spanish-speaking breast cancer prevention Latinas American study to ensure it was culturally appropriate [49]. This indicates a recognition of the unique knowledge of the impacted study population and how this may aid the intervention; however, further details on how the study population engaged in the research process were not provided.

Greater involvement of the impacted population in the research process was achieved in five studies. These studies appeared to use a form of academic–community partnership with defined responsibilities for both partners, providing the benefit of a diversity of perspectives which can result in stronger solutions to health problems [58]. The US study with American Indian women used weekly planning meetings to design the intervention and then met monthly during implementation stages. The study found that shared perspectives on how best to reduce alcohol consumption led to a broader wellness intervention with an integrated cultural approach [51]. Similarly, high levels of participation were evident in the mHealth RCT involving the study population in funding acquisition, research agenda setting, study design, and dissemination of research findings [52]. The highest level of power sharing between academic researchers and communities was evident in the three community coalition Thailand-based projects [53,54,55], with communities involved in problem definition, intervention development, and study design through community forums.

### 3.4. Enablers for Intervention Effectiveness

Working with communities as partners in the research process appeared to support an increase in self-efficacy and social supports for participants. Mobilisation, “power sharing”, and intervention design by women for women were enablers for effectiveness. The high levels of engagement of PAR where participants contributed to research decision-making enabled enhanced social support. The incorporation of unique knowledge about the challenges the local community faced may have contributed to the intervention effectiveness through improved dietary intake and reductions in alcohol use [49,51].

Key enablers for success were attributed to the PAR approach where the study population worked with researchers. This “power sharing” approach, with local women involved in the research process, was credited with building capacity and enabling effective peer-led health promotion [49,51,54,55]. The capacity-building workshops increased self-esteem, confidence, and assertiveness, which allowed women to access and share information with their families and community. The participatory approach also allowed for a root cause of the issue, gender equality and alcohol, to be addressed [55].

Interventions designed by the study population, for the study population, created a welcoming, familiar, and nurturing setting. This improved engagement with the intervention and the mechanism of increased social support and self-confidence may have contributed to the positive outcomes of reducing alcohol use and improving fruit and vegetable intake [48,49,51,52,54]. Likewise, the benefits of incorporating culture were found through creating a safe space to discuss culturally sensitive topics related to women’s health and were credited with increasing confidence and self-efficacy [49,51].

### 3.5. Challenges to Interventions

The context the intervention was placed into, intervention upscaling, and community-based research were the main challenges identified. The involvement of multiple communities in the research decision-making process led to time delays when studies were at a greater scale, as seen in the multi-community regional-sized study [53]. However, this challenge was not seen across the single community interventions despite the active involvement by multiple community and academic groups in the research process [48,49,50,51,52,54,55]. For the upscaled regional study, the disadvantages to the PAR approach increased timeframes, which had flow-on effects [53]. The delayed initiation of prevention activities meant the intervention length was shortened for some activities, and this may have reduced the effect [53]. The RCT study design resulted in a loss of flexibility to adapt to this context. Important to note for future interventions is that other studies with RCT study designs and single-community focus did not suffer from this challenge [48,50,52].

The methodological challenges included the appropriateness of interventions to the political context, the need to maintain research–community partnerships, and contamination of control groups. The structurally focused intervention incorporating policy changes found it was difficult to maintain research–community partnerships over a decade or more, the time needed to see the effects of structural changes [53]. Additionally, the synergy of the intervention with the political context must be considered. A time of civil unrest and a political agenda of law-enforcement approach to drug enforcement was at odds with the harm-reduction approach of the intervention [53]. It is hard to contain interventions in a community-based context, and potential “ripple effects” may have tempered comparisons between groups in several studies [50,51,53].

## 4. Discussion

This systematic review of peer-reviewed studies evaluating participatory approaches to breast cancer prevention lifestyle interventions and alcohol identified a small pool of literature with eight articles. Despite the small and heterogenous literature with methodological limitations due to study designs, this represents the best available evidence. Seven of the eight interventions that used PAR reported positive results. The findings highlight the potential of PAR, particularly for alcohol interventions with women, and illuminate how interventions can be designed for maximum impact. The participatory research combined the efforts of the impacted study population with researchers as partners in the research process. The collaboration with shared decision-making, agenda setting, intervention design, development of research tools, and analysis were all areas that researchers undertook with communities. However, the participation levels varied between studies, which may have impacted the effectiveness of interventions. 

The intervention enablers of self-confidence, social support, and community mobilisation identified in this review are congruent with previous participatory research in chronic disease [19]. The findings of increased self-confidence to enact positive behaviour change, increased connection through the strengthening of social networks, and the increased capacity for community action have been identified as the mechanisms of intervention effectiveness in previous research and in this review [19,48,49,51,54,55]. The aspects of improved intervention delivery and the longer-term benefits of community development were found in previous cardiovascular disease participatory research and in this review through the studies with community coalition approaches [53,54,55]. A key enabler of participatory research through improved intervention design through the input of the impacted population was congruent with previous research [18,49,51,52].

Given the research demonstrating elevated cancer risk with alcohol use, there is a clear need for more research in this area. Participatory research approaches offer the opportunities for communities, clinicians, and researchers to work together, and can improve research translation, providing sustainable outcomes. The benefits of participatory research approaches offer the opportunity to mobilise the community and to create tailored strategies which meet the needs of the targeted population. Positive results were reported in seven of the eight studies [48,49,50,51,52,54,55], and the use of participatory approaches were credited by several authors as contributing to effectiveness. Future interventions should consider the evaluation of levels of self-confidence for behavioural change, perceived strength of social networks, and the capacity of the community to mobilise and take action in their designs. 

The reviewed literature included a single study on diet-focused breast cancer prevention and seven alcohol interventions. However, any intervention which reduces alcohol consumption may have the secondary benefit of breast cancer prevention. The use of participatory research in this field is clearly still in infancy. The reviewed literature provides evidence that women were receptive and responsive to the approach, and their buy-in is essential for addressing socially entrenched behaviours, such as alcohol consumption. Future research should consider the benefits of collaboration with the impacted population in the research process. 

### Review Strengths and Limitations

The strength of the review is the synthesis of quantitative literature about the effect of interventions with the qualitative understanding of how the individuals and communities benefitted [59]. The incorporation of different types of knowledge improved the utility of the review to inform future interventions through what worked and how. The limitations of the review are similar to other PAR reviews [24] where only empirical studies published in academic journals were included. This decision was made to increase the methodological rigor and facilitate inclusion of higher quality studies; however, this may have resulted in publication bias of positive results, which could result in an overly optimistic conclusion on the effectiveness of PAR. The definition of PAR principles is not clear-cut and there is debate within the literature. To define PAR in a way that could be consistently applied in the screening process, the involvement of the study population in the research process was used utilising a definition by a previous participatory review; however, some researchers would argue that this is not in line with pure participatory action research. Given the emerging state of the literature, it was decided there was pragmatic value in discussing the varied ways of engaging the study population and how this impacted the research process, rather than taking a purist ideological stance. The emerging state of the literature meant there was a high degree of heterogeneity across the studies. The diversity in terms of study designs, outcomes, populations, and settings of the included studies meant that the effectiveness of these studies could be attributed to many factors besides the participatory approach. This reduced our ability to draw conclusions about the effectiveness of PAR. However, the studies provide important methodological lessons on how researchers can benefit from engaging the study population in the research process in the primary prevention of breast cancer. 

## 5. Conclusions

This systematic review highlights the potential for participatory research in breast cancer prevention and the mechanisms that support effectiveness. Previous studies have found participatory approaches beneficial through incorporating the voices of the impacted population. The participatory research approach provides an opportunity to partner with those who have lived experience. The benefits of the PAR approach were the diverse perspectives brought to the research process, which influenced agenda setting, intervention design, and evaluation. The partnership between academics and communities allowed for more flexible and culturally tailored solutions to the barriers commonly faced in seeking to improve diet or reduce alcohol consumption. 

Strong evidence exists regarding alcohol as a risk factor for breast cancer; however, no current research on breast cancer prevention interventions intervening on alcohol through participatory approaches was found. It is recommended that new interventions integrate the key lessons of utilising participatory approaches to realize the benefits of stronger engagement and increasing self-confidence and enhancing social networks. These mechanisms were important enablers for effective lifestyle change outcomes. Considerations for the synergy of the intervention with the political climate, the need for balancing flexibility and rigor in study designs, and to avoid overreaching with the scale of implementation are important to reduce challenges. The findings provide evidence that women were receptive and responsive to the participatory approach, and their buy-in is essential for addressing socially entrenched behaviours, such as alcohol consumption. Participatory research presents opportunities for future interventions to intervene on the modifiable risk factors for breast cancer.

## Figures and Tables

**Table 1:** Inclusion and exclusion criteria.

Criterion	Inclusion	Exclusion
**Time period**	2008 to May 2021	Studies prior to 2008
**Study design**	Qualitative, mixed-method, and quantitative empirical studies published in peer-reviewed scientific journals.	Literature not published in peer-reviewed scientific journals.
**Intervention focus**	Intervention with participatory action research principles where the study population participated in at least one stage of agenda setting, research planning, data collection, analysis, or data interpretation.	Observational studies or studies with no evidence of participatory action research principles, for example, where the study population are passive participants.
**Outcomes**	Study reports on the outcomes or impact of the program on the study population related to the behaviour, perception, knowledge, risk, or attitudes of drinking alcohol or breast cancer prevention risk factors (smoking, physical activity, diet).	Exclude studies reporting outcomes solely on screening rates, surgical, genetic, pharmacological, or alcohol dependence/disorder.
**Population**	Human participants with a mean age greater than 18 years.	Animal studies or studies with male-only participants or participants diagnosed with breast cancer or where the majority are veterans (armed forces current or have previously served) or prisoners or homeless people or preconception-focused or pregnant women or sex workers or emergency/trauma acute care setting interventions.

**Table 2:** Characteristics of included studies.

Project Name	Risk Factor	Population and Setting	Paradigm	Study Design	Intervention and Select Results
Nuestra Cocina: Cancer Risk Reduction Intervention [49]	Nutrition	Women at community health centres	Quantitative	Quasi-experimental	The academic-community partnership intervention found improvements in breast cancer prevention knowledge and dietary improvements.
Storytelling 4 Empowerment Group [52]	Alcohol	Young people at community health centres	Quantitative	Randomized control trial (RCT)	The mHealth tablet-based application intervention found reductions in alcohol use compared to the control group.
Preventing Rural Thai Methamphetamine Abuse and HIV by Community Mobilization [53]	Alcohol	Young people in multiple settings; rural primary schools, community health/hospital and community settings	Quantitative	RCT	The community coalition intervention found that alcohol use remained high in control and intervention communities. Only minor changes were present from baseline, and the overall study reported a null effect.
Community-developed health promotion intervention [51]	Alcohol	Women at a house in their local neighbourhood	Quantitative	Quasi-experimental	The culturally-focused wellness intervention found reductions in alcohol consumption with women consuming moderate to high levels of alcohol prior to the intervention achieving greater substantial absolute decrease than lighter drinkers.
Peer Mentoring for Individuals With Traumatic Brain Injury and Their Significant Others [48]	Alcohol	Women and men at a rehabilitation hospital	Quantitative	RCT	Significant reductions in alcohol use were found in the intervention group compared to the control.
Brief Alcohol Screening and Intervention for College Students [50]	Alcohol	Young people at a college campus	Quantitative	RCT	Significant reductions in alcohol use were found in the experimental group from baseline to 3-month follow-up.
Family Health Leader project in north eastern Iaan region of Thailand [55]	Alcohol	Women in community forums and home visits	Qualitative	Quasi-experimental	Peer modelling was effective in reducing alcohol consumption through empowering participants to share knowledge regarding alcohol use and providing family support for members with problematic alcohol consumption.
Family Health Leader project north eastern Loie region of Thailand [54]	Alcohol	Women and men in community forums and home visits	Qualitative	Quasi-experimental	Peer-led interventions led to significant decrease in AUDIT (Alcohol use Disorder Identification Test) scores compared to pre-intervention levels.

## Appendix A

Search strategy for Ovid—Medline, PsycInfo, Emcare
**Alcohol**
exp alcohol drinking/ or alcohol$ or
(alcohol adj2 (problem or pattern or burden or freq* or injury or dependen* or disorder* or drink* or misuse or excess or harm or binge or hazard* or abuse* or consum* or use* or behaviour or behavior or excess* or reduc* or cessation or intoxicat*)).
**Breast cancer**
breast neoplasms or (breast tumor* or breast tumour* or mammary carcinoma* or human mammary car-cinomas or mammary neoplasms or cancer of the breast or cancer of breast).
**Prevention**
primary prevention/ or secondary prevention/ or (prevent* or control*).
**Intervention**
((program or randomi* or placebo or random* or trial* or pre or post or before or after or intervention or experiment* or evaluat* or quasi).mp. or (nonequivalent control group or posttesting or pretesting or pretest posttest design or pretest posttest control group design or quasi experimental methods or quasi experimental study or time series or time series analysis).sh. or (((nonequivalent or non equivalent) adj3 control$) or posttest$ or post test$ or pre test$ or pretest$ or quasi experiment$ or quasiexperiment$ or timeseries or time series).mp.) not (observational or case-control or case control or cross-sectional or cross sectional or case report or case series).
**PAR**
Community-Based Participatory Research/ or cooperative inquiry.mp. or appreciative inquiry.mp. or action learning.mp. or Cooperative Behavior/ or cooperative behaviour.mp. or knowledge trans*.mp. or implementation science.mp. or research co-production.mp. or collaborative research.mp. or knowledge mobilization.mp. or knowledge mobilisation.mp. or paradigm science.mp. or citizen science.mp. OR
(codesign* or co-design* or coproduc* or co-produc* or cocreat* or co-creat* or codevelop* or collaborative design or participatory or e-collaboration or Experience based design or experience-based design or EBCD or ((user* or patient* or consumer* or participant* or client* or stakeholder* or peer* or communit* or decision-maker or female or women or citizen*) adj2 (centre* or center* or centric or involv* or participat* or partner* or activat* or advisor* or engag* or collaborat* or consult* or empower* or input* or led or focus* or develop* or plan* or deliver* or servic* or program or strat*))) OR
(Social justice or participatory or PAR or action research or Community-driven or community driven or consumer driven or Consumer-driven or Citizen Science or paradigm science or user-led or consumer-panel or advisory board or community health promotion).
All searches were limited to 2008–2021; no language restriction was applied.
